# Association between sleep quality and central obesity among southern Chinese reproductive-aged women

**DOI:** 10.1186/s12905-021-01407-0

**Published:** 2021-08-04

**Authors:** Bingbing Li, Nan Liu, Donghui Guo, Bo Li, Yan Liang, Lingling Huang, Xiaoxiao Wang, Zhenzhen Su, Guozeng Zhang, Peixi Wang

**Affiliations:** 1 Institute of Chronic Disease Risks Assessment, School of Nursing and Health, Henan University, Kaifeng, 475004 China; 2 Nursing Department, Tongji Hospital, Tongji Medical College, Huazhong University of Science and Technology, Wuhan, 430030 Hubei Province China; 3 General Practice Center, The Seventh Affiliated Hospital, Southern Medical University, Foshan, PR China; 4 The People’s Hospital of Longhua.Shenzhen, Shenzhen, 518109 China; 5 The Affiliated Hospital of Xuzhou Medical University, Xuzhou, 221006 China; 6 Institute of Nursing and Health, Shanghai Lida University, Shanghai, 201609 China

**Keywords:** Sleep quality, Central obesity, Reproductive-aged women, Pittsburgh Sleep Quality Index, Chinese

## Abstract

**Background:**

The connections between sleep quality and central obesity among reproductive-aged women are not clear. The study aimed to explore the association between sleep quality and central obesity among Chinese reproductive-aged women and identify the independent contributions of sociodemographic characteristics, health-related factors, and sleep quality to central obesity.

**Methods:**

In this cross-sectional survey, the minimal sample sizes were 2404 subjects; 2449 Chinese women aged 18–49 participated in this study. Sleep quality was assessed by the Chinese version of the Pittsburgh Sleep Quality Index (PSQI). Central obesity as the outcome of interest was a binary variable; women were categorized as with versus without central obesity measured by waist circumference (WC). The independent contribution of sociodemographic characteristics (Cluster 1), health-related variables (Cluster 2), and sleep quality (Cluster 3) to central obesity was derived from the corresponding *R*^2^ change (individual *R*^2^ change/total *R*^2^ × 100%), using clustered multiple logistic regression analyses.

**Results:**

The risk of central obesity increased significantly with poor sleep quality (assessed by global PSQI score) [adjusted odds ratio (OR) = 2.20 per SD increase; 95% confidence interval (CI) = 1.28–3.78; *P* = 0.004], increased sleep disturbance score (adjusted OR = 1.11 per SD increase; 95% CI = 1.01–1.22; *P* = 0.042) and decreased subjective sleep quality score (adjusted OR = 0.81 per SD increase; 95% CI = 0.73–0.90; *P* < 0.001). The independent contribution of sleep quality was 9.9%, less than those of sociodemographic (73.3%) and health-related (16.8%) variables. Among complaints related to sleep disturbance, the inability to breathe comfortably, and having bad dreams showed significant associations with central obesity.

**Conclusions:**

There exists some degree of correlation between sleep quality and central obesity among Chinese reproductive-aged women. These findings underscore the need for future public health guidelines to formulate some detailed strategies to improve sleep quality, such as preventing and intervening risk factors that influence sleep quality and suggesting optimal sleep duration, which might effectively reduce the incidence of central obesity in this population group.

## Background

Obesity is a global public health issue estimated to be majorly responsible for increased mortality from cardiovascular diseases and cancers [1, 2]. Moreover, obesity, especially central obesity reflecting the abdominal adipose tissue, which is effectively evaluated by waist circumference (WC), is reported to be correlated with insulin resistance and is also recognized as a crucial risk factor for the development and progression of metabolic syndrome (MetS) [3]. It is necessary to understand the innumerable factors that could affect the occurrence and development of obesity.

Currently, sleep is considered as a major role to maintain health. Sleep restriction can result in impaired metabolism and endocrine function [4, 5]. Short or long sleep duration and poor sleep quality have been reported to be correlated with both general and central obesity [6–8]. However, the association of sleep including duration and quality with obesity are not consistent [9, 10].

It is noteworthy that most conclusions on the sleep-obesity relationship were derived from studies by using body mass index (BMI) as the main indicator of obesity. BMI, as an adiposity index, is generally used to reflect the extent of peripheral obesity; nevertheless, another obesity type, central obesity is ignored. As numerous studies have verified, central obesity is strongly correlated with the development of various obesity-related chronic diseases and is better reflected by measuring WC [11–13]. Recent analyses demonstrated that the prevalence of central obesity was 29.6% among Chinese women [14]; the trend is continuing to escalate worldwide, including in China [15, 16]. It has also been demonstrated that central obesity was negatively associated with asthma control [17], and positively associated with infertility [18], insulin resistance [19], hypertension [20], rheumatoid arthritis [21], subclinical myocardial dysfunction [22], and depressive symptoms of females [23]. Hence, determining the underlying causes as well as the potentially modifiable risk factors is crucial to improving prevention program design, and decreasing the prevalence of central obesity among women.

Females are more prone to complain about sleep problems than males [24]. Poor sleep quality among women may make them more vulnerable to obesity and other diseases, which can seriously affect their fitness and quality of life. Furthermore, reproduction might be also affected. Still, the association between sleep quality and central obesity amongst Chinese reproductive-aged women remains uncertain. Therefore, the study aims to use clustered logistic regression to analyze potential associations between sleep quality (measured by the PSQI) and central obesity (as determined by WC) and to identify the independent contribution of sleep quality to central obesity in Chinese reproductive women aged 18–49 years. Analyzing these associations may enhance our knowledge concerning the relationship between sleep quality and central obesity of Chinese reproductive-aged females. Additionally, it may support the discovery of novel strategies to prevent central obesity and promote the health of this population.

## Methods

### Study design

This cross-sectional health survey was conducted in a district of the Pearl River Delta region of China in 2018, through face-to-face interviews at participants’ residences.

### Study population and sampling

We selected 2513 reproductive-aged females by simple random sampling. According to the sample size, the number of participants in the survey was calculated proportionally in each community and health service center could provide the household lists. Participants were then randomly selected from the household lists.

### Study population and sampling

The prevalence of central obesity among women was 35.0% in our pre-survey in the area; with a maximal tolerance at 2.0% and error of type I at 5%, the minimal sample sizes were 2185 reproductive-aged females. Considering 10% invalid sample size (10% non-response rate), at least 2404 women with the age of 18–49 years were needed.

### Inclusion and exclusion criteria

Non-pregnant or lactating women between the ages of 18 and 49 years were included. Those with a history of sleep-related breathing disorders (e.g., sleep apnea–hypopnea syndrome (OSAHS), obesity hypoventilation syndrome (OHS), and others), restless legs syndrome, depression, psychiatric disorders, cancer, ovariectomy, postmenopause, estrogen therapy, and others were excluded.

### Study variables

#### Measurement of sleep quality

The PSQI of a translated version was used to assess sleep quality. It is a standard self-report 19-item questionnaire designed to collect the subjective nature of one’s sleep habit over 1 month [25]. Each item with a four-point scale ranges from 0 to 3. In many settings, the PSQI has been used in diagnosing sleep disorders and has proven to have good reliability and validity [26–28]. It estimates several different aspects of sleep, which reflect seven aspects of sleep problems, including subjective sleep quality, sleep latency, sleep duration, habitual sleep efficiency, sleep disturbance, use of sleep medication, and daytime dysfunction [25]. Their sum constitutes the global sleep quality score (ranging from 0 to 21), with a higher score indicating worse sleep quality. A global PSQI score above seven differentiates poor from good sleepers, with high diagnostic sensitivity and specificity (98.3% and 90.2%, respectively) in the Chinese population [29].

### General research questionnaire

The questionnaire included the following variables: sociodemographic characteristics (age group, marital status, educational level, and occupational status), health-related factors (smoking, drinking, exercise, hypertension, diabetes mellitus, 2-week morbidity, and hospitalization in the last year), and sleep quality (assessed by the PSQI). The definition of smoking was having smoked more than 100 cigarettes in their lifetime. The definition of drinking was having consumed alcohol of more than 30 g/week within the last 12 months. The exercise was categorized into three statuses based on the frequency (No exercise, 1–2 times/week, and ≥ 3 times/week). Self-reported information on doctor-diagnosed hypertension/diabetes mellitus, 2-week morbidity, and hospitalization in the last year was obtained. The questionnaire has previously been described in a study [30]. Central obesity was measured by WC. Qualified examiners, including physicians and nurses, obtained the WC (to the nearest 0.1 cm) by duplicate-measuring the midpoint between the edge of the lower rib and the iliac crest and averaged the values [31]. Central obesity was defined according to the recommendations of the International Diabetes Federation definition of MetS as WC ≥ 80 cm in women [32].

### Statistical analysis

Data analyses were performed using SPSS 18.0 (SPSS, Inc., Chicago, IL, USA). Descriptive statistics were used to summarize participant characteristics. Bivariate analyses using independent samples *t*-test and χ^2^ test were performed to draw a comparison between groups. Multivariate analysis was performed by using clustered logistic regression analyses (forward stepwise method), where central obesity was considered as the dependent variable and sociodemographic characteristics, health-related factors, and sleep quality were independent variables. Before the multivariate analysis, we first analyzed the multicollinearity among the overall sleep quality and seven domains of the PSQI. In the multicollinearity analysis for independent variables, values of the variance inflation factor (VIF) were all lower than 2.0, which demonstrated that there were no multicollinearity problems. Then, in the multivariate analysis, both the overall sleep quality and seven domains of PSQI were included in one model in Cluster 3. A two-sided 0.05 statistical significance level was set for all analyses. During the regression analysis, PSQI component scores as continuous variables were standardized for better comparison.

**Fig. 1:**
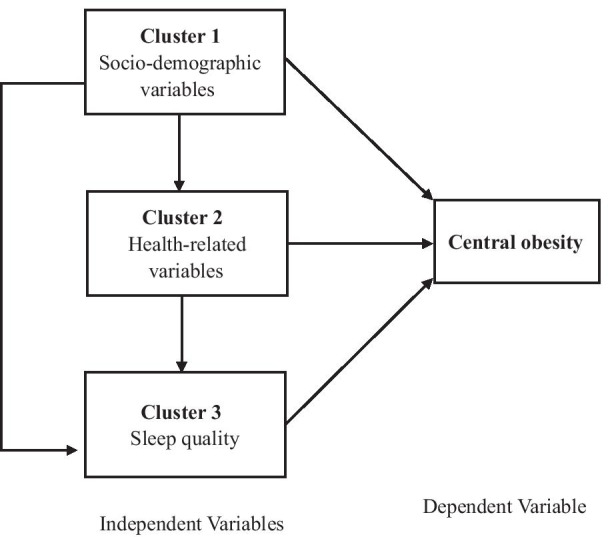
The clustered multiple logistic regression model and multi-directional associations (the direction of the impact is indicated by the direction of the arrows)

Specifically, the independent contributions of sociodemographic characteristics, health-related variables, and sleep quality to central obesity were assessed using clustered multiple logistic regression analyses [33, 34]. There was the possibility of multi-directional links between the three clusters and the dependent variable as shown in Fig. 1. Cluster 1 could have affected Clusters 2 and 3 as well as the dependent variable. Similarly, Cluster 2 might have affected Cluster 3 and the dependent variable. However, Cluster 3 might only have influenced the dependent variable. Consequently, variables in the prior cluster might have an impact on variables in the subsequent cluster [24]. We determined the final regression model in three phases, the details were shown in previous studies [33].

The independent effect of each cluster was derived from the corresponding *R*^2^ change, which resembled the classical *R*^2^ applied to linear regression models [34]. Then the formula (individual *R*^2^ change/total *R*^2^ × 100%) was used to calculate the independent contribution of each cluster [34].

### Ethical considerations

Written informed consent was obtained from each study participant. The People's Hospital of Longhua. Shenzhen Medical Ethics Committee approved the study (2917055).

## Results

### Respondent characteristics

A total of 2449 women participated in this survey. The average age was 33.0 ± 7.5 years (SD), with roughly 32.4% reproductive-aged women with central obesity. Most participants were married (81.9%), employed (79.2%), and had completed high school or higher education (78.4%).

### Bivariate analysis

As age increased, there was a higher prevalence of central obesity (*P* < 0.001). Also, the prevalence of hypertension (*P* < 0.001), diabetes mellitus (*P* = 0.005) and hospitalization in the last year (*P* < 0.001) was significantly related to central obesity in women. Scores of seven PSQI components were described as mean + SD, varying from 0.02 ± 0.17 to 0.55 ± 0.66, in which the mean component score of use of sleep medication was the lowest, while the component of sleep latency was scored the highest. More details of participants’ characteristics are presented in Tables 1 and 2.

**Table 1:** Prevalence of central obesity by characteristics

Variable	Total N = 2449 (%)	Central obesity		*P* value
		Yes, 794 (%)	No, 1655 (%)	
Cluster 1: sociodemographic				
Age group (years)				< 0.001
18–25	380 (15.5)	57 (15.0)	323 (85.0)	
26–35	1217 (49.7)	397 (32.6)	820 (67.4)	
36–45	674 (27.5)	261 (38.7)	413 (61.3)	
46–49	178 (7.3)	79 (44.4)	99 (55.6)	
Marital status				< 0.001
Unmarried	403 (16.5)	61 (15.1)	342 (84.9)	
Married	2005 (81.9)	720 (35.9)	1285 (64.1)	
Widowed	9 (0.4)	1 (11.1)	8 (88.9)	
Divorced	32 (1.2)	12 (37.5)	20 (62.5)	
Educational level				< 0.001
Primary school or lower	86 (3.5)	47 (54.7)	39 (45.3)	
Middle school	444 (18.1)	170 (38.3)	274 (61.7)	
High school or above	1919 (78.4)	577 (30.1)	1342 (69.9)	
Occupational status				< 0.001
Employed	1940 (79.2)	598 (30.8)	1342 (69.2)	
Retired	14 (0.6)	6 (42.9)	8 (57.1)	
Student	89 (3.6)	15 (16.9)	74 (83.1)	
Unemployed	406 (16.6)	175 (43.1)	231 (56.9)	
Cluster 2: health-related				
Smoking				0.498
Yes	9 (0.4)	2 (22.2)	7 (77.8)	
No	2440 (99.6)	792 (32.5)	1648 (67.5)	
Drinking				0.111
Yes	13 (0.5)	7 (53.8)	6 (46.2)	
No	2436 (99.5)	787 (32.3)	1649 (67.7)	
Exercise				0.802
Over 3 times/week	824 (33.6)	260 (31.6)	564 (68.4)	
1–2 times/week	794 (32.4)	262 (33.0)	532 (67.0)	
No exercise	831 (33.9)	272 (32.7)	559 (67.3)	
Hypertension				< 0.001
Yes	29 (1.2)	21 (72.4)	8 (27.6)	
No	2420 (98.8)	773 (31.9)	1647 (68.1)	
Diabetes mellitus				0.005
Yes	9 (0.4)	7 (77.8)	2 (22.2)	
No	2440 (99.6)	787 (32.3)	1653 (67.7)	
2-week morbidity				0.523
Yes	66 (2.7)	19 (28.8)	47 (71.2)	
No	2383 (97.3)	775 (32.5)	1608 (67.5)	
Hospitalization in the last year				< 0.001
Yes	154 (6.3)	75 (48.7)	79 (51.3)	
No	2295 (93.7)	719 (31.3)	1576 (68.7)	
Cluster 3: PSQI domain scores				
Global PSQI score				0.005
≤ 7	2373 (96.9)	758 (31.9)	1615 (68.1)	
> 7	76 (3.1)	36 (47.4)	40 (52.6)

**Table 2:** Comparison between central obesity and non-central obesity groups

Variable	Total N = 2449 (M ± SD)	Central obesity		*P* value
		Yes, 794 (M ± SD)	No, 1655 (M ± SD)	
Cluster 3: PSQI domain scores				
Subjective sleep quality	0.42 ± 0.60	0.41 ± 0.61	0.43 ± 0.59	0.417
Sleep latency	0.55 ± 0.66	0.55 ± 0.68	0.54 ± 0.65	0.716
Sleep duration	0.38 ± 0.52	0.40 ± 0.56	0.37 ± 0.50	0.167
Habitual sleep efficiency	0.15 ± 0.47	0.15 ± 0.49	0.14 ± 0.46	0.544
Sleep disturbance	0.33 ± 0.49	0.37 ± 0.52	0.31 ± 0.48	0.008
Use of sleep medication	0.02 ± 0.17	0.03 ± 0.21	0.02 ± 0.15	0.217
Daytime dysfunction	0.31 ± 0.64	0.32 ± 0.68	0.31 ± 0.62	0.609

*M* mean, *SD* standard deviation

### Global and component PSQI scores and central obesity

Poor sleep quality was significantly associated with central obesity [crude odds ratio (OR) = 2.76; 95% confidence interval (CI) = 1.49–5.14; *P* = 0.001] (Table 3). The risk of central obesity was significantly increased with the elevated component PSQI score in sleep disturbance (crude OR = 1.17; 95% CI = 1.06–1.29; *P* = 0.001) and the decreased component PSQI score in subjective sleep quality (crude OR = 0.86; 95% CI = 0.77–0.96; *P* = 0.005).

**Table 3:** Associations between PSQI components and central obesity among females (N = 2449)

PSQI components^a^	Crude OR^b^	95% CI	*P* value
Global PSQI score (≤ 7)	1	Reference	
> 7	2.76	1.49–5.14	0.001^†^
Subjective sleep quality	0.86	0.77–0.96	0.005^†^
Sleep latency	0.96	0.88–1.06	0.46
Sleep duration	1.60	0.97–1.16	0.21
Habitual sleep efficiency	0.95	0.86–1.05	0.27
Sleep disturbance	1.17	1.06–1.29	0.001^†^
Use of sleep medication	1.05	0.97–1.14	0.21
Daytime dysfunction	0.96	0.86–1.06	0.40

*CI* confidence interval

^†^*P* < 0.05

^a^The seven sleep quality domains and global PSQI score (as a dichotomous variable) were included as predictor variables for central obesity in a regression model without adjustment for variables in Clusters 1 and 2

^b^Odds ratio per standard deviation increase in a predictor variable

### Multivariate analysis

#### Influential factors of central obesity

**Fig. 2:**
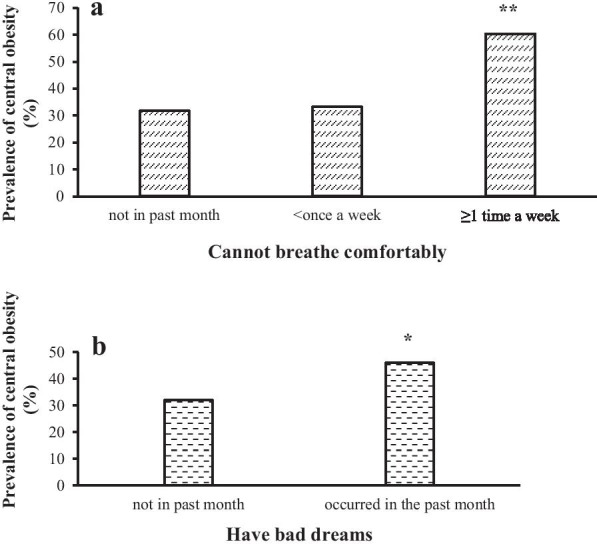
Comparisons of the prevalence of central obesity in participants with sleep disturbances. **P < 0.01, *P < 0.05, after adjustment for sociodemographic variables in Cluster 1 and health-related variables in Cluster 2; (**a**) ≥ 1 time a week compared with not in the past month, *P* = 0.001; ≥ 1 time a week compared with < once a week, *P* = 0.003; (**b**) occurred in the past month compared with not in the past month, *P* = 0.040

In Cluster 1, age group, marital status, educational level, and occupational status were associated with central obesity (Table 4). Their independent contribution was 73.3%. In Cluster 2, hypertension, diabetes mellitus, and hospitalization in the last year were positively associated with central obesity. Their independent contribution was 16.8%. In Cluster 3, the risk of central obesity increased significantly with poor sleep quality (adjusted OR = 2.20; 95% CI = 1.28–3.78; *P* = 0.004), increased sleep disturbance score (adjusted OR = 1.11; 95% CI = 1.01–1.22; *P* = 0.042), and decreased subjective sleep quality score (adjusted OR = 0.81; 95% CI = 0.73–0.90; *P* < 0.001). The independent contribution of the third cluster to central obesity was 9.9%. Moreover, associations between complaints of sleep disturbances and central obesity were further analyzed and illustrated in Fig. 2. Among the symptoms of sleep disturbances, the inability to breathe comfortably and having bad dreams showed significant associations with central obesity. Participants who were unable to breathe comfortably more than once a week had considerably higher odds of central obesity than those who did not have this problem and those who had experienced it less than once a week in the last month (*P* = 0.001 and 0.003). The prevalence of central obesity in participants who have experienced bad dreams was significantly higher than in those who had not in the past month (*P* = 0.040). In addition, women in the survey claiming the highest category of subjective sleep quality (very good) obtained higher odds of central obesity than those as “fairly good” (*P* < 0.001).

**Table 4:** Cluster logistic regression models explaining central obesity by variables in three clusters

Predictor variable^a^	OR^b^ (95% CI)	*P*	Nagelkerke *R*^2c^	Independent contribution (%)
Cluster 1				
Age group (years) (15–25)	Reference			
26–35	2.04 (1.40–2.98)	< 0.001		
36–45	2.47 (1.65–3.68)	< 0.001		
46–49	2.58 (1.59–4.19)	< 0.001		
Marital status (unmarried)	Reference			
Married	1.90 (1.31–2.74)	0.001		
Educational level (primary school or lower)	Reference			
High school or above	0.48 (0.30–0.77)	0.002		
Occupational status (employed)	Reference			
Unemployed	1.40 (1.11–1.77)	0.004		
Total			0.074	73.3%
Cluster 2				
Hypertension, yes versus no	4.17 (1.78–9.75)	0.001		
Diabetes mellitus, yes versus no	5.32 (1.07–26.45)	0.041		
Hospitalization in the last year, yes versus no	1.88 (1.33–2.65)	< 0.001		
Total			0.091	16.8%
Cluster 3				
Global PSQI score, > 7 versus ≤ 7	2.20 (1.28–3.78)	0.004		
Subjective sleep quality	0.81 (0.73–0.90)	< 0.001		
Sleep disturbance	1.11 (1.01–1.22)	0.042		
Total			0.101	9.9%

The forward stepwise method was used in the logistic regression analysis

^a^Only variables with *P* ≤ 0.05 were included in the model

^b^For age group, marital status, educational level, occupational status, hypertension, diabetes mellitus, hospitalization in the last year, and sleep quality domain scores, odd ratios per standard deviation increase were presented

^c^Nagelkerke *R*^2^ in this study is the variance of the dependent variable (central obesity), which could be explained by variables in three clusters included in the regression model

## Discussion

The purpose of this study was to explore the association between sleep quality and central obesity and to evaluate the effects of sleep quality on central obesity among Chinese reproductive-aged women. Our analyses revealed that poor sleep quality was significantly associated with central obesity. The odds of central obesity were significantly positive with the sleep disturbance scores and negatively related to subjective sleep quality scores. In addition, the symptoms of sleep disturbances (inability to breathe comfortably and having bad dreams) were associated with central obesity. The independent contribution of sleep quality to central obesity was smaller than those of sociodemographic and health-related variables.

Our study identified sleep quality as a significant independent determinant of central obesity among Chinese reproductive-aged women. Poor sleep quality was significantly associated with central obesity after adjusting for variables in Clusters 1 and 2. A population-based German study, including 753 adults aged 35–65 years, indicated that poor sleep quality was associated with obesity and high body fat mass [9]. In an earlier study among African Americans, Bidulescu reported that the global sleep quality score was only related to obesity in females [35].

Several potential mechanisms accounting for the link of poor sleep quality and central obesity might be due to reduced sleep quality impacting physiological changes with metabolism, hormone secretion, and appetite regulation. These changes could lead to energy dysregulation that results in obesity. For instance, the secretion of ghrelin and leptin and neuronal activity in response to food stimuli are proved to be regulated by sleep restriction and act on appetite regulation, thus affecting food choice and calorie intake [4, 36]. Besides, poor sleep quality can be linked to sleepiness and fatigue. It may lead to reduced daytime physical activity indirectly, thus encouraging weight gain and abdominal fat accumulation [37, 38]. The reduction in physical activity seems to be a plausible pathway by which poor sleep quality could result in obesity. Overall, this kind of sleep-induced disbalance in energy intake and expenditure might play a crucial part in weight changes. The relations between sleep quality and central obesity have not been fully understood and the mechanisms of these possibilities are needed to be substantiated in future longitudinal or follow-up studies.

In the current study, we found that women with higher scores in the sleep disturbance component had a higher prevalence of central obesity after controlling for sociodemographic (Cluster 1) and health-related (Cluster 2) factors. These results were also observed in a study conducted on 796 Taiwanese male police officers [39]. Similarly, another study reported that to some extent, females with elevated sleep disturbance scores had greater odds of abdominal obesity than those without [6]. Furthermore, we found that among the symptoms of sleep disturbances, the inability to breathe comfortably and having bad dreams showed significant associations with central obesity. As such, women with sleep disturbances should be screened for central obesity to prevent obesity-related chronic diseases. However, this tends to be problematic since the sleep-obesity link might be two-way. Although poor sleep could predispose one to obesity by altering energy regulatory hormones and behaviors, obesity is also likely to exacerbate sleep problems via co-occurring sleep-related breathing disorders [40]. Thus, our findings may help guide improvements with screening methods for unrecognized sleep-related breathing disorders in groups with complaints of sleep disturbance (inability to breathe comfortably). In addition, stress-induced systemic inflammation might be a biological mechanism linking sleep disturbances, central obesity, and other chronic diseases [41]. Women at reproductive age (15–49 years old), might face multiple pressures from work, family, economy, and society. The presence of sleep disturbances in this population group might be caused by stress, while at the same time, sleep disturbances could also bring about stress. This vicious circle would undoubtedly seriously affect women's health. Therefore, good sleep quality is critical to holistic health, including maintaining a healthy body shape for women.

Interestingly, we found that women reporting the highest category of subjective sleep quality (very good) had higher odds of central obesity than women reporting it as “fairly good” (after controlling for variables in Clusters 1 and 2). A U-shaped correlation of total sleep duration with BMI has been reported in some prior studies, especially in women [7, 8]. One possible explanation for this finding is that individuals who have very good subjective sleep quality might have longer sleep durations and thus have relatively less time during the day to engage in physical activity, which could serve as an indirect mechanism linking very good subjective sleep quality with central obesity. In our study, there was certainly a slight difference in average sleep duration between women who reported “very good” and “fairly good” subjective sleep quality categories (7.8 h per night vs. 7.6 per night; *P* < 0.001). Besides sleep quality, this finding might provide supporting evidence in highlighting the importance of optimal sleep duration for preventing central obesity among women. More studies are necessary to elucidate potential mediators of the relationship.

We also found that the independent contribution of sleep quality to central obesity was smaller than those of sociodemographic and health-related factors. Obesity is the result of a fat accumulation over a period of time. Yet, the PSQI reflects sleep quality for the last month, which may be an insufficient period of time for assessing the effect of sleep quality on central obesity. It would, therefore, be worthwhile to consider a longer follow-up to further assess the impact of sleep quality on central obesity. Although the concrete mechanisms linking poor sleep quality and central obesity are not fully clear, the results do suggest that sleep quality is correlated with WC in Chinese reproductive-aged women. In light of central obesity which has been proved to be fairly relevant to chronic and metabolic diseases, reasonable sleep might be of benefit in the prevention and intervention of obesity.

Among the three clusters, the first cluster made the greatest independent contribution to central obesity, and age was significantly associated with central obesity. The previous study has found that aging is related to raised adiposity in white adipose tissues as well as thermogenic impairment in brown adipose tissue, which may increase the incidence of obesity [42]. Besides, in females, estrogen receptor (ER) α was proven to have a protective role in maintaining metabolic homeostasis [43]. Earlier studies have shown that there was an inverse link between age and gene expression for ERα and the ratio of ERα to ERβ in female abdominal subcutaneous fatty tissue [44]. Some researchers have found that the prevalence of obesity varies not only by age but also by income and educational levels [45, 46]. Consistently, in our study, unemployed participants had higher odds of central obesity than those employed ones; we also observed that compared with women who had primary school or lower education, those who had an educational level of high school or above had lower odds of central obesity. It might be due to cognitive skills and health literacy varying among individuals in different educational levels. To prevent central obesity of reproductive-aged women and promote their health, further studies are necessary to clarify the specific mechanisms between the relevant factors and central obesity among this population group.

## Limitations and strengths

Several limitations of this study should be noted. First, due to the cross-sectional nature of the study, the causality of sleep quality and central obesity might not be elucidated; longitudinal and intervention studies may provide a better understanding about it. Second, we did not measure hormones or laboratory indexes (e.g., ghrelin, leptin, insulin resistance, or sympathoadrenal activity), which might be the mediator between sleep quality and central obesity. Thirdly, some key factors (e.g., frequency, times, types, and amounts of meals) that might provide detailed information in explaining the relationships between sleep quality and central obesity were not included. Lastly, there might be reporting or recall bias for some of the variables (e.g. the smoking, drinking, exercise variables, etc.) in this study and too few subjects met the criteria of the smoking status.

Reproductive-aged women as a special population group, our results demonstrated for the first time an independent association of sleep quality and central obesity in this population group in China. The independent contribution of sleep quality to central obesity was calculated and the associations between them were revealed. This study could bring new data to the field of sleep quality and central obesity-related reproductive-aged women’s health, and underscore findings previously reported by similar studies from other geographical regions.

## Conclusion

There exists some degree of associations between sleep quality and central obesity among Chinese reproductive-aged women. Poor sleep quality and the components of subjective sleep quality and sleep disturbance (inability to breathe comfortably and having bad dreams) were associated with central obesity. These findings underscore the need for future public health guidelines to formulate some detailed strategies to improve sleep quality, such as preventing and intervening risk factors that influence sleep quality and suggesting optimal sleep duration, which might effectively reduce the incidence of central obesity in this population group.

## Data Availability

The data supporting our findings are kept in confidentiality and available from the corresponding author on reasonable request.

## Abbreviations

PSQI: The Pittsburgh Sleep Quality Index
CI: Confidence interval
OR: Odds ratio
MetS: Metabolic syndrome
WC: Waist circumference
SAHS: Sleep apnea–hypopnea syndrome
ER: Estrogen receptor
